# Diagnostic Value of Serum miR-182, miR-183, miR-210, and miR-126 Levels in Patients with Early-Stage Non-Small Cell Lung Cancer

**DOI:** 10.1371/journal.pone.0153046

**Published:** 2016-04-19

**Authors:** WangYu Zhu, KaiYu Zhou, Yao Zha, DongDong Chen, JianYing He, HaiJie Ma, XiaoGuang Liu, HanBo Le, YongKui Zhang

**Affiliations:** 1 Laboratory of Cytobiology and Molecular Biology, Zhoushan Hospital of Wenzhou Medical University, Zhoushan, Zhejiang, 316021, China; 2 Department of Cardio-Thoracic Surgery, Zhoushan Hospital of Wenzhou Medical University, Zhoushan, Zhejiang, 316021, China; 3 Lung Cancer Research Center, Zhoushan Hospital of Wenzhou Medical University, Zhoushan, Zhejiang, 316021, China; Penn State University, UNITED STATES

## Abstract

Blood-circulating miRNAs could be useful as a biomarker to detect lung cancer early. We investigated the serum levels of four different miRNAs in patients with non-small cell lung cancer (NSCLC) and assessed their diagnostic value for NSCLC. Serum samples from 112 NSCLC patients and 104 controls (20 current smokers without lung cancer, 23 pneumonia patients, 21 gastric cancer patients, and 40 healthy controls) were subjected to Taqman probe-based quantitative reverse transcription–polymerase chain reaction (RT-PCR). The data showed that the serum levels of miR-182, miR-183, and miR-210 were significantly upregulated and that the miR-126 level was significantly downregulated in NSCLC patients, compared with the healthy controls. Further receiver operating characteristic (ROC) curve analysis revealed that the serum miR-182, miR-183, miR-210, or miR-126 level could serve as a diagnostic biomarker for NSCLC early detection, with a high sensitivity and specificity. The combination of these four miRNAs with carcinoembryonic antigen (CEA) further increased the diagnostic value, with an area under the curve (AUC) of 0.965 (sensitivity, 81.3%; specificity, 100.0%; and accuracy, 90.8%) using logistic regression model analysis. In addition, the relative levels of serum miR-182, miR-183, miR-210, and miR-126 could distinguish NSCLC or early-stage NSCLC from current tobacco smokers without lung cancer and pneumonia or gastric cancer patients with a high sensitivity and specificity. Data from the current study validated that the four serum miRNAs could serve as a tumor biomarker for NSCLC early diagnosis.

## Introduction

Lung cancer is a leading cause of cancer-related deaths in the world, and up to 85% of lung cancer is classified as non-small cell lung cancer (NSCLC) [1]. To date, more than 75% of NSCLC patients are diagnosed at advanced or metastatic stages of disease, which results in a poor overall survival rate of less than 15% [1]. Non-resectable NSCLC also leads to a high rate of tumor recurrence after chemo-radiation therapy, thus contributing to a poor survival [1]. In contrast, the five-year survival rate of patients with pathological stage IA NSCLC currently reaches to 83.9% and that of stage IB NSCLC is approximately 66.3% [2]. Thus, early detection is still the key to improve the survival of NSCLC patients. To date, the diagnosis of early-stage NSCLC mainly relies on computed tomography, magnetic resonance imaging, positron emission tomography, sputum cytology, or histological examination of bronchoscopic tissues [3]. These techniques are expensive or generally not practical for NSCLC early detection in a large cohort of the population. Thus, the development of a noninvasive or minimally invasive blood biomarker, such as blood-circulating DNA [4], methylated genes [5], tumor cells, or microRNAs (miRNAs), could provide a novel approach for the early clinical detection of NSCLC. Moreover, the development of more stable, sensitive, and specific biological markers could provide an easy to use and accurate tool for clinicians to diagnosis cancer earlier than is currently possible.

miRNA is a class of non-coding small RNAs of up to 24 nucleotides in length that is very stable in the blood circulation; therefore, it could be useful for NSCLC detection, providing a high sensitivity and specificity [6, 7]. Indeed, several previous studies have demonstrated that the levels of circulating miRNAs are altered in patients with NSCLC, compared with those in healthy individuals [8–12]. For example, Mitchell *et al*. [8] in 2008 first reported that serum miR-141 is upregulated in prostate cancer, suggesting that it could distinguish prostate cancer patients from healthy controls. In addition, Markou *et al*. [9] have described altered levels of three circulating miRNAs (i.e., miR-21, miR-10a, and miR-30e-5p) in NSCLC patients and demonstrated their potential utility in the detection of NSCLC. Other studies further analyzed the sensitivity and specificity of different miRNAs in the diagnosis of early-stage NSCLC [6, 10]. For instance, Tang *et al*. [11] have demonstrated that the combination of miR-21, miR-155, and miR-145 was a suitable biomarker to distinguish NSCLC patients from controls as it yielded a sensitivity of 76.5%, a specificity of 81.3%, and an area under the curve (AUC) of 0.87. An additional study evaluated the dynamics of serum miR-21 and miR-24 in pre- and post-operative NSCLC patients as biomarkers to predict treatment effectiveness [12]. Overall, the use of a panel of miRNAs increases the sensitivity and specificity in the differential diagnosis of early-stage NSCLC, since miRNA expression is altered in a range of diseases including cancer, infectious diseases, or even healthy individuals who smoke tobacco. Therefore, in this study, we validated these four miRNAs as being specifically dysregulated in the sera of NSCLC patients [9, 13–15] and explored their value in the early and differential diagnosis of the disease. This study demonstrates that the use of a set of miRNAs provides a useful diagnostic tool for the early diagnosis of NSCLC.

## Materials and Methods

### Patients

A total of 221 blood samples meeting eligibility criteria were collected from Zhoushan Hospital, Zhejiang Province, China, between January 2011 and September 2014. These samples included 112 NSCLC patients, 20 current smokers without lung cancer, 23 patients with pneumonia, 21 patients with gastric cancer, and 40 healthy controls. Lung and gastric cancers were diagnosed histologically and further confirmed by two pathologists, according to the National Comprehensive Cancer Network criteria [16, 17]. Patients with pneumonia were diagnosed based on the clinical manifestations, sputum bacterial culture, and radiographic findings. All patients had no prior history of therapy, including surgery, chemotherapy, radiotherapy, or antibiotic therapy. Patients and control subjects were free of other concomitant diseases, such as chronic obstructive pulmonary disease, diabetes, hypertension, asthma, hepatitis, tuberculosis, and neurological or psychiatric disorders. Table 1 summarizes the clinicopathological characteristics of the study subjects. This study was approved by the Ethics Review Committee of Zhoushan Hospital of China. Written informed consent was obtained from all of the participants. All participants were ethnic Han Chinese.

**Table 1:** Clinicopathological characteristics of the subjects in this study n (%).

Clinical features	NSCLC (n = 112)	Pneumonia (n = 23)	Gastric cancer (n = 21)	Smoking individuals (n = 20)	Healthy controls (n = 40)
Mean age (years)	58.5 ± 11.6	63.9 ± 15.1	59.9 ± 7.9	57.0 ± 4.5	57.9 ± 6.8
< 60	61 (54.5)	8 (34.8)	9 (42.9)	5 (25.0)	25 (62.5)
≥ 60	51 (45.5)	15 (65.2)	12 (57.1)	15 (75.0)	15 (37.5)
Gender					
Male	60 (53.6)	14 (60.9)	16 (76.2)	20 (100.0)	22 (55.0)
Female	52 (46.4)	9 (39.1)	5 (23.8)	0 (0.0)	18 (45.0)
Tobacco smoking					
None	72 (64.3)	7 (30.4)	9 (42.9)	0 (0.0)	40 (100.0)
Current	40 (35.7)	16 (69.6)	12 (57.1)	20 (100.0)	0 (0.0)
Tumor size (cm)					
≤ 2	78 (69.6)	-		-	-
> 2	34 (30.4)	-		-	-
Histology					
Adenocarcinoma	90 (80.4)	-	21 (100.0)	-	-
SCC	22 (19.6)	-	0 (0.0)	-	-
Lymph node metastasis					
Negative	95 (84.8)	-	11 (52.4)	-	-
Positive	17 (15.2)	-	10 (47.6)	-	-
Tumor stage					
0	5 (4.5)	-	-	-	-
IA, IB	82 (73.2)	-		-	-
IIA, IIB	15 (13.4)	-		-	-
IIIA, IIIB	10 (8.9)	-		-	-

Blood samples were collected from all participants early in the morning. Sera were immediately separated after blood collection and stored at -80°C until use. The laboratory technicians were blinded to the patient’s identity. A chemiluminescence method was used to measure miRNA in the sera. As part of the routine clinical assessment, data pertaining to carcinoembryonic antigen (CEA) levels were ascertained from the patients’ medical records from Zhoushan Hospital.

### Serum Preparation, RNA Isolation, and Quantitative Reverse Transcription–Polymerase Chain Reaction (RT-PCR)

To obtain serum samples, 10 mL of peripheral blood was drawn into separate gel tubes and then subjected within 30 min to centrifugation at 1,500 *g* for 10 min at 4°C. The supernatants were transferred to 1.5-mL tubes and stored at -80°C until use. To detect miRNA expression, 600 μL of the serum sample from each participant was subjected to RNA isolation using a mirVana PARIS RNA isolation kit (Applied Biosystems, Foster City, CA, USA), according to the manufacturer’s protocol. The RNA concentration was determined using a NanoDrop ND-1000 spectrophotometer (NanoDrop Technologies) and a 15% denatured polyacrylamide gel. The RNA samples were subjected to a reverse transcription reaction using a TaqMan MicroRNA Reverse Transcription Kit (Applied Biosystems), according to the manufacturer’s instructions. Subsequently, qPCR was carried out on the serum samples in triplicate using TaqMan 2× Universal PCR Master Mix with no AmpErase UNG (Applied Biosystems) on an ABI 7500 Real-Time PCR system (Applied Biosystems). qPCR amplification conditions were set to an initial cycle of 95°C for 10 min followed by 40 cycles of 95°C for 15 s and 60°C for 1 min. The cycle threshold (Ct) values were calculated using SDS 2.0.1 software (Applied Biosystems). No template controls were used in either the RT or PCR steps to ensure target-specific amplification. The control miRNA was U6 small nuclear RNA (snRNA), according to a previous study [18]. The average expression levels of serum miRNA were calculated using the 2^-ΔCt^ method relative to the average of U6 snRNA [19]. To determine the fold change of target miRNAs relative to miRNAs expressed in normal controls, the expression 2^-ΔΔCt^ was used [20]. The mean Ct value of the four miRNAs was calculated, excluding outliers (i.e., replicates with Ct differing by more than one cycle from the median). In addition, if the mean Ct value for U6 was not between 20 and 32 cycles, the assay was repeated at least once on some of the samples. Samples with low U6 snRNA levels were not included for data analysis in this study.

### Statistical Analysis

Statistical analyses were performed with GraphPad Prism 5.0 software (GraphPad Software Inc., San Diego, CA) and MedCalc 9.0 software (MedCalc Software Inc., Mariakerke, Belgium). The data were examined according to the degree of homogeneity. The unpaired *t* test or Mann-Whitney test was used to analyze the differences between subjects and controls or the correlation between miRNA expression levels and patient clinicopathological features. Receiver operating characteristic (ROC) curves were generated to assess the diagnostic accuracy of each parameter. The best sensitivity/specificity pair was selected based on the maximum Youden Index. The AUC values were also calculated and compared using a nonparametric approach [21]. Multiple logistic regression analysis was performed to assess the diagnostic accuracy of the combination of serum miRNAs in NSCLC patients. All statistical tests were two-sided, and a *P* value ≤ 0.05 was considered statistically significant.

## Results

### Differential Expression of Serum miRNAs in Different Diseases and Healthy Controls

**Fig 1:**
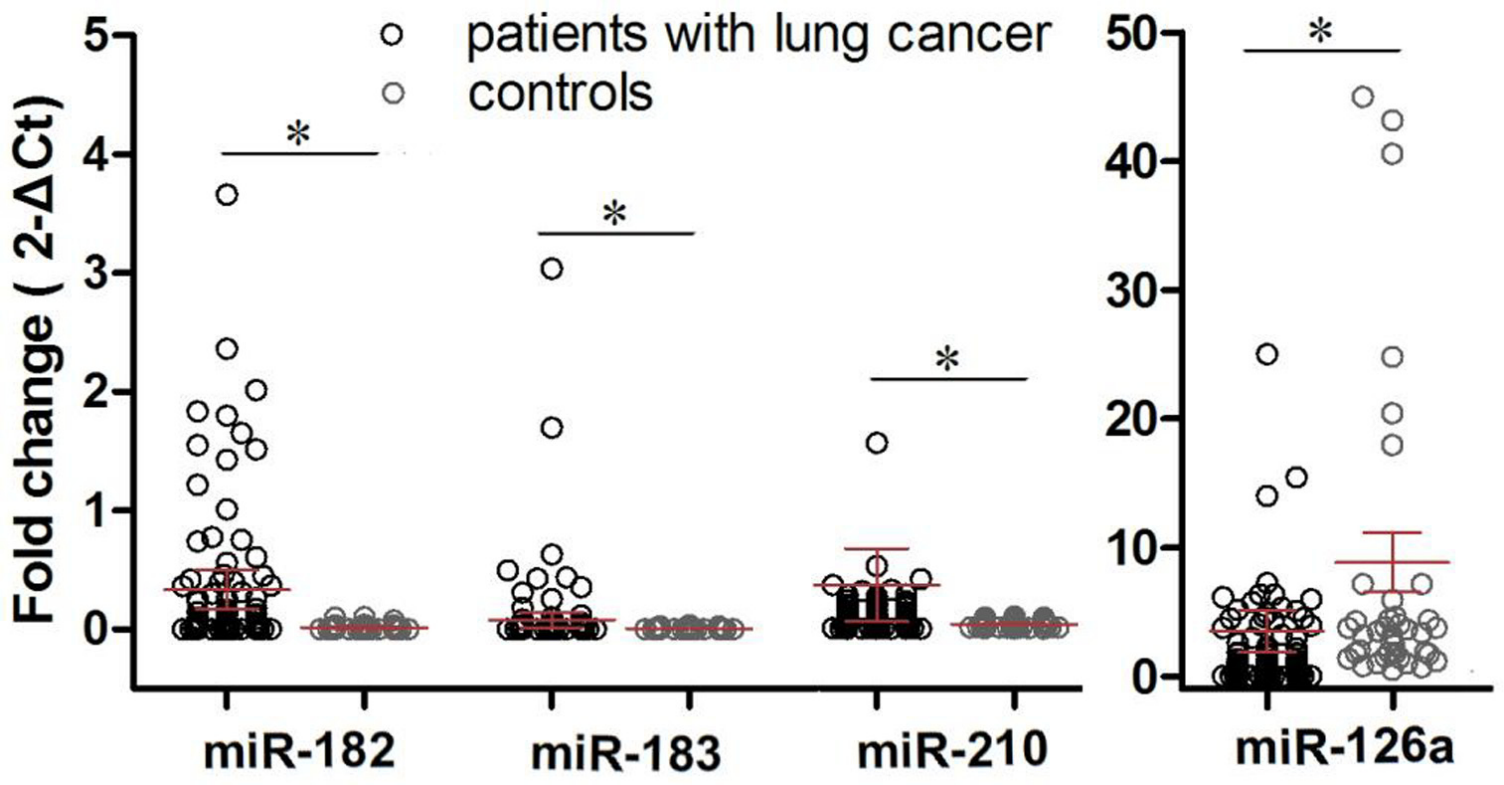
Expression of miR-182, miR-183, miR-210, and miR-126 in sera from NSCLC patients and healthy controls. Graphs show dot-plots of medians and inter-quartile ranges of log_2_-transformed values of each miRNA in sera from 112 NSCLC patients (*black*) and 40 healthy controls (*grey*). U6 snRNA was used as the reference. *P* values of miR-182, miR-183, miR-210, and miR-126 were *<* 0.0001, 0.0181, 0.0299, and < 0.0001, respectively, using the Mann-Whitney test. *P < 0.05 between patients and controls.

Based on previous studies [9, 13–15, 22], we selected and assessed four dysregulated serum miRNAs in 216 samples (i.e., 112 NSCLC patients, 23 pneumonia patients, 21 gastric cancer patients, 20 current smokers without lung cancer, and 40 healthy controls). Our data showed that the levels of miR-182, miR-183, and miR-210 were significantly upregulated in the sera from NSCLC patients, compared to those of age- and gender-matched healthy controls (*P <* 0.0001, 0.0181, and 0.0299, respectively), whereas the serum miR-126 levels were significantly decreased in the NSCLC patients (*P <* 0.0001; Fig 1).

### Circulating Serum miRNA Distinguishes NSCLC Patients from Pneumonia Patients, Gastric Cancer Patients, and Tobacco Smokers

We found that the relative levels of serum miR-183 and miR-210 expression were increased in the NSCLC patients, compared to those of smokers (*P <* 0.0001 and 0.0024, respectively). The serum levels of miR-182, miR-183, and miR-210 were also increased in the NSCLC patients, compared to those of the pneumonia patients (*P* = 0.0197, 0.0400, and 0.0278, respectively); whereas the serum level of miR-182 was decreased in the NSCLC patients, compared to that in the gastric cancer patients. In addition, the serum level of miR-183 was increased in the NSCLC vs. gastric cancer patients (*P <* 0.0001 and 0.0351, respectively).

**Fig 2:**
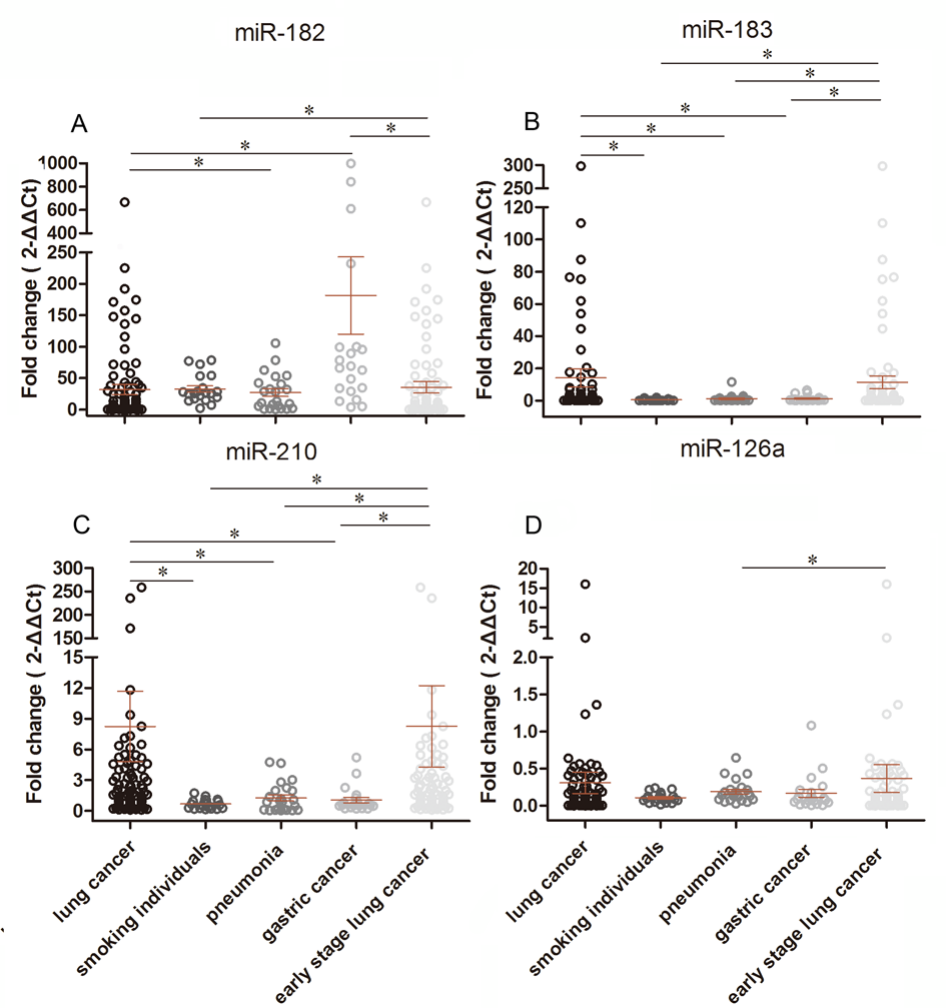
Serum levels of miRNAs in NSCLC or early-stage NSCLC patients versus smokers, pneumonia patients, and gastric cancer patients. The serum levels of miR-182, miR-183, miR-210, and miR-126 were assessed in 112 NSCLC patients, 20 current smokers, 23 pneumonia patients, and 21 gastric cancer patients using qRT-PCR. U6 snRNA was used as the reference. *P < 0.05 between groups using the Mann-Whitney test.

In 87 early-stage (0 and I) NSCLC patients, the serum levels of miR-182, miR-183, and miR-210 were significantly increased, compared to those of smokers (*P* = 0.0015, < 0.0001, and 0.0006, respectively); the serum levels of miR-183 and miR-210 were also increased, whereas the miR-126 level was decreased, compared with the pneumonia patients (*P* = 0.0293, 0.0142, and 0.0113, respectively). The serum levels of miR-183 and miR-210 were also increased and the serum level of miR-182 was decreased, compared to those of the gastric cancer patients (*P* = 0.0239, 0.0048, and < 0.0001, respectively; Fig 2). However, there was no obvious difference in the serum levels of these four miRNAs (miR-182, miR-183, miR-210, and miR-126) between early-stage and late-stage NSCLC patients (*P* = 0.0724, 0.2874, 0.2991, and 0.4754, respectively).

### Early Detection Value of the Four Serum miRNAs for NSCLC Patients vs. Healthy Controls

**Fig 3:**
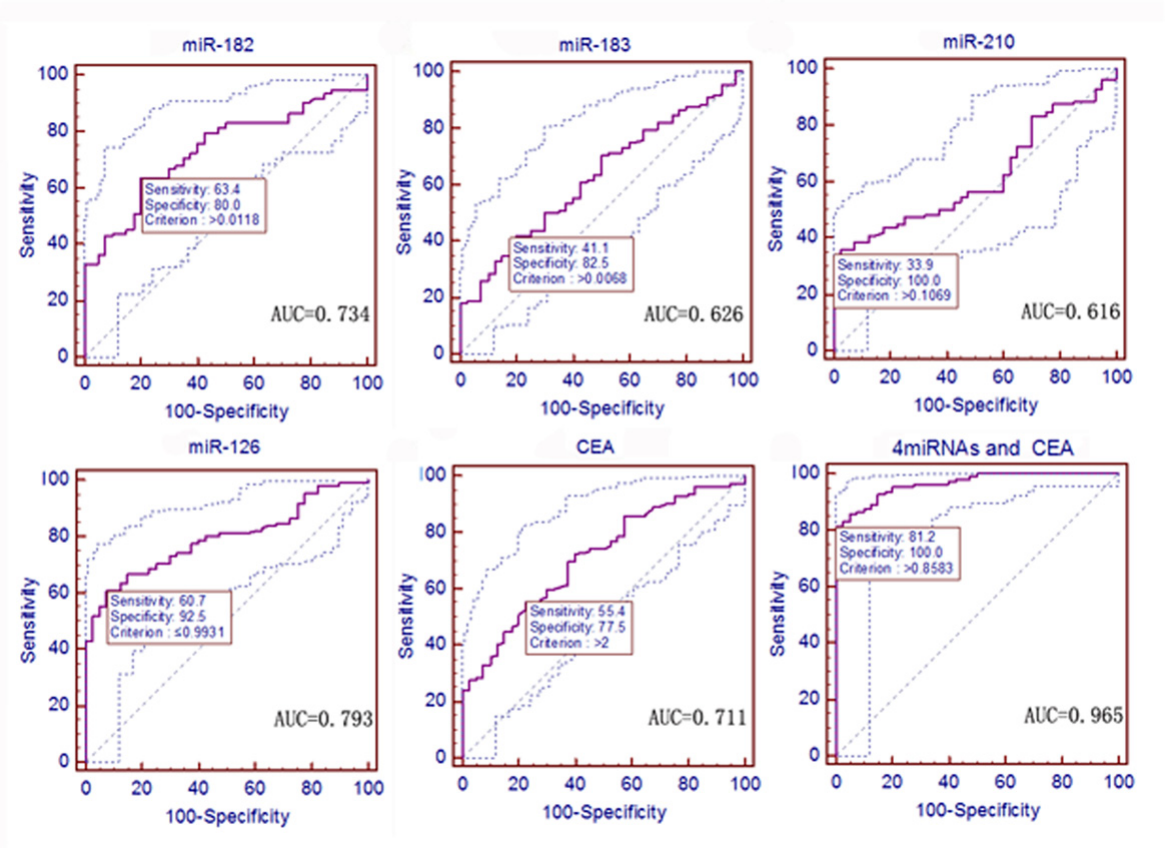
ROC curves to assess the value of serum miRNA and CEA levels in NSCLC patients compared to 40 healthy controls. The *P* values of serum miR-182, miR-183, miR-210, miR-126, and CEA as well as the predictive value of logistic regression were *<* 0.0001, 0.0091, 0.0121, < 0.0001, < 0.0001, and < 0.0001, respectively.

Next, we performed ROC curve analysis to evaluate the diagnostic value of these four serum- circulating miRNAs and CEA. First, we identified a cut-off value that distinguished 112 NSCLC patients from 40 healthy sex- and age-matched controls. The ROC curve data showed that all four of these miRNAs could distinguish the NSCLC patients from the controls. Specifically, the serum level of miR-182 had a sensitivity of 63.4% and a specificity of 80.0% to differentiate the NSCLC patients from the healthy controls, with an AUC of 0.734 at the optimal cut-off point (*P <* 0.0001, 95% confidence interval [CI]: 0.657–0.803). miR-183 had a sensitivity of 41.1% and a specificity of 82.5%, with an AUC of 0.626 (*P* = 0.0091, 95% CI: 0.544–0.703). miR-210 had a sensitivity of 33.9% and a specificity of 100.0%, with an AUC of 0.616 (*P* = 0.0121, 95% CI: 0.534–0.694). miR-126 had a sensitivity of 60.7% and a specificity of 92.5%, with an AUC of 0.793 (*P <* 0.0001, 95% CI: 0.719–0.854). CEA had a sensitivity of 55.4% and a specificity of 77.5%, with an AUC of 0.711 (*P <* 0.0001, 95% CI: 0.632–0.782; Fig 3). Clinically, the serum CEA level is routinely used for cancer detection. Thus, we compared the AUC of CEA with that of the four miRNAs and found that the diagnostic value of CEA was not significantly different from those of miR-182, miR-183, miR-210, and miR-126 (*P* = 0.7290, 0.2056, 0.1818, and 0.1271, respectively). However, the predicted values of logistic regression analysis showed that the combined ROC analysis of these four miRNAs plus CEA revealed an increased AUC value of 0.965, with a sensitivity of 81.3%, a specificity of 100.0%, and an accuracy of 90.8% (*P* < 0.0001, 95% CI: 0.923–0.988; Fig 3 and Table 2). The multivariate logistic regression analysis of the ROC curve of these four miRNAs plus CEA is shown in S1 Table.

**Table 2:** Sensitivity, specificity, and areas under the curves (AUC) of the four miRNAs plus CEA in NSCLC or early-stage NSCLC vs. normal controls (2^-ΔCt^).

Potential tumor marker	Cut-off value	Sensitivity (%)	Specificity (%)	AUC (95% CI)	*P*
NSCLC					
miR-182	0.0118	63.4	80.0	0.734 (0.657–0.803)	*<* 0.0001
miR-183	0.0068	41.1	82.5	0.626 (0.554–0.703)	0.0091
miR-210	0.1069	33.9	100.0	0.616 (0.534–0.694)	0.0121
miR-126	0.9931	60.7	92.5	0.793 (0.719–0.854)	*<* 0.0001
CEA	2.0	55.4	77.5	0.711 (0.632–0.782)	*<* 0.0001
The four miRNAs + CEA	0.8583	81.2	100.0	0.965 (0.923–0.988)	*<* 0.0001
Early-stage NSCLC					
miR-182	0.0118	67.8	85.0	0.781 (0.699–0.849)	*<* 0.0001
miR-183	0.0068	41.4	82.5	0.638 (0.548–0.721)	0.0065
miR-210	0.1069	35.6	100.0	0.650 (0.561–0.721)	0.0019
miR-126	0.9931	62.1	97.5	0.845 (0.770–0.903)	*<* 0.0001
CEA	1.74	63.2	62.5	0.648 (0.558–0.731)	0.0045
The four miRNAs + CEA	0.6736	88.5	92.5	0.975 (0.930–0.994)	*<* 0.0001

**Fig 4:**
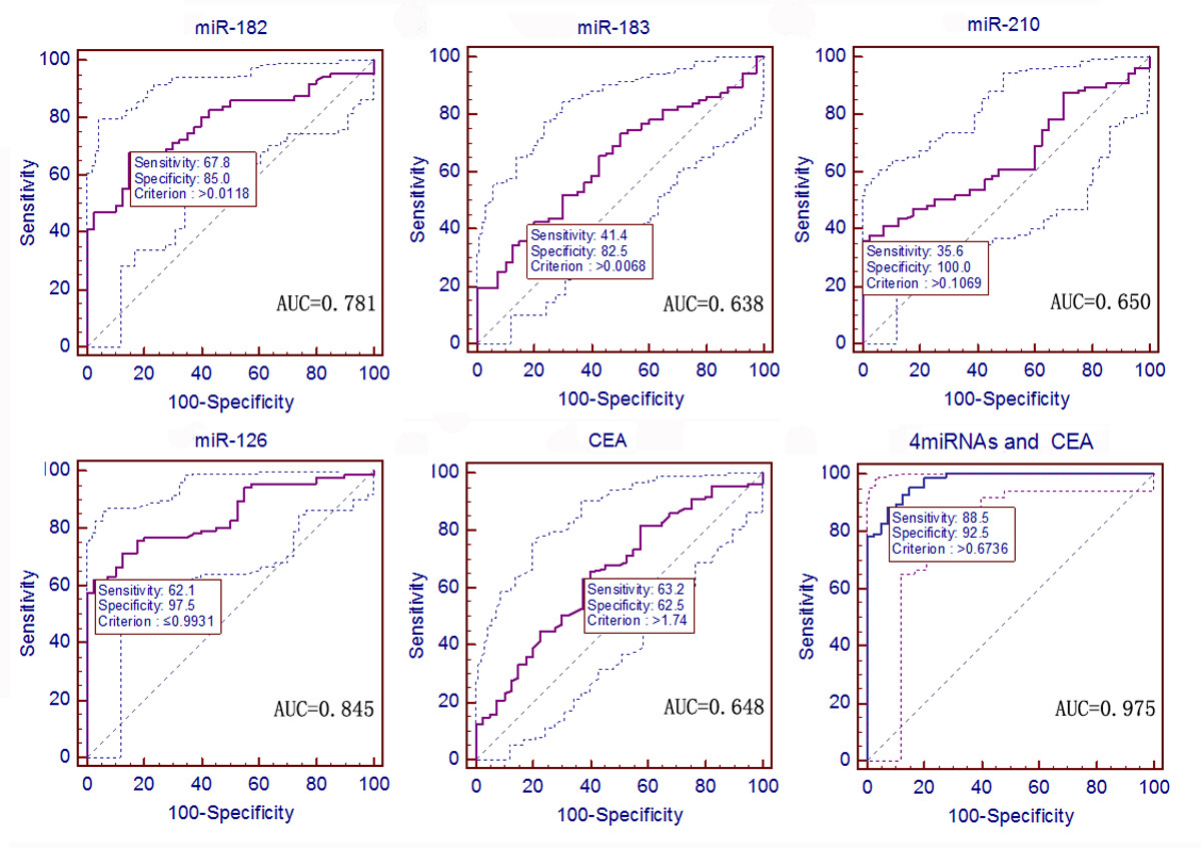
ROC curves to assess the value of serum miRNA and CEA levels in stage 0 and I NSCLC patients compared to 40 healthy controls. The *P* values of the serum level of miR-182, miR-183, miR-210, miR-126, and CEA as well as the predictive value of logistic regression were < 0.0001, 0.0065, 0.0019, < 0.0001, 0.0045, and < 0.0001, respectively.

Moreover, ROC curve analysis was used to assess the diagnostic value of these four miRNAs between 87 early-stage NSCLC (stage 0 and I) patients and 40 sex- and age-matched healthy controls. The data showed that the serum levels of miR-182 had an AUC of 0.781, with a sensitivity of 67.8% and a specificity of 85.0% (*P <* 0.0001, 95% CI: 0.699–0.849); miR-183 had an AUC of 0.638, with a sensitivity of 41.4% and a specificity of 82.5% (*P* = 0.0065, 95% CI: 0.548–0.721); miR-210 had an AUC of 0.650, with a sensitivity of 35.6% and a specificity of 100.0% (*P* = 0.0019, 95% CI: 0.561–0.721); miR-126 had an AUC of 0.845, with a sensitivity of 62.1% and a specificity of 97.5% (*P <* 0.0001, 95% CI: 0.770–0.903); while CEA had an AUC of 0.648, with a sensitivity of 63.2% and a specificity of 62.5% (*P* = 0.0045, 95% CI: 0.558–0.731; Fig 4). The AUC of miR-126 was wider compared with that of CEA (*P* = 0.0012). Nevertheless, the AUCs of miR-182, miR-183, and miR-210 were not different from the AUC of CEA (*P* = 0.0589, 0.8855, and 0.9796, respectively). In addition, the predicted values of the logistic regression analysis showed that the combined ROC analysis of these four miRNAs plus CEA displayed an increased AUC value of 0.975, with a sensitivity of 88.5%, a specificity of 92.5%, and an accuracy of 91.3% (*P <* 0.0001, 95% CI: 0.930–0.994; Fig 4 and Table 2). Multivariate logistic regression analysis of the ROC curve of these four miRNAs and CEA is shown in S1 Table.

### Diagnostic Value of These Four Serum miRNAs plus CEA in NSCLC Patients vs. Other Disease Groups

**Fig 5:**
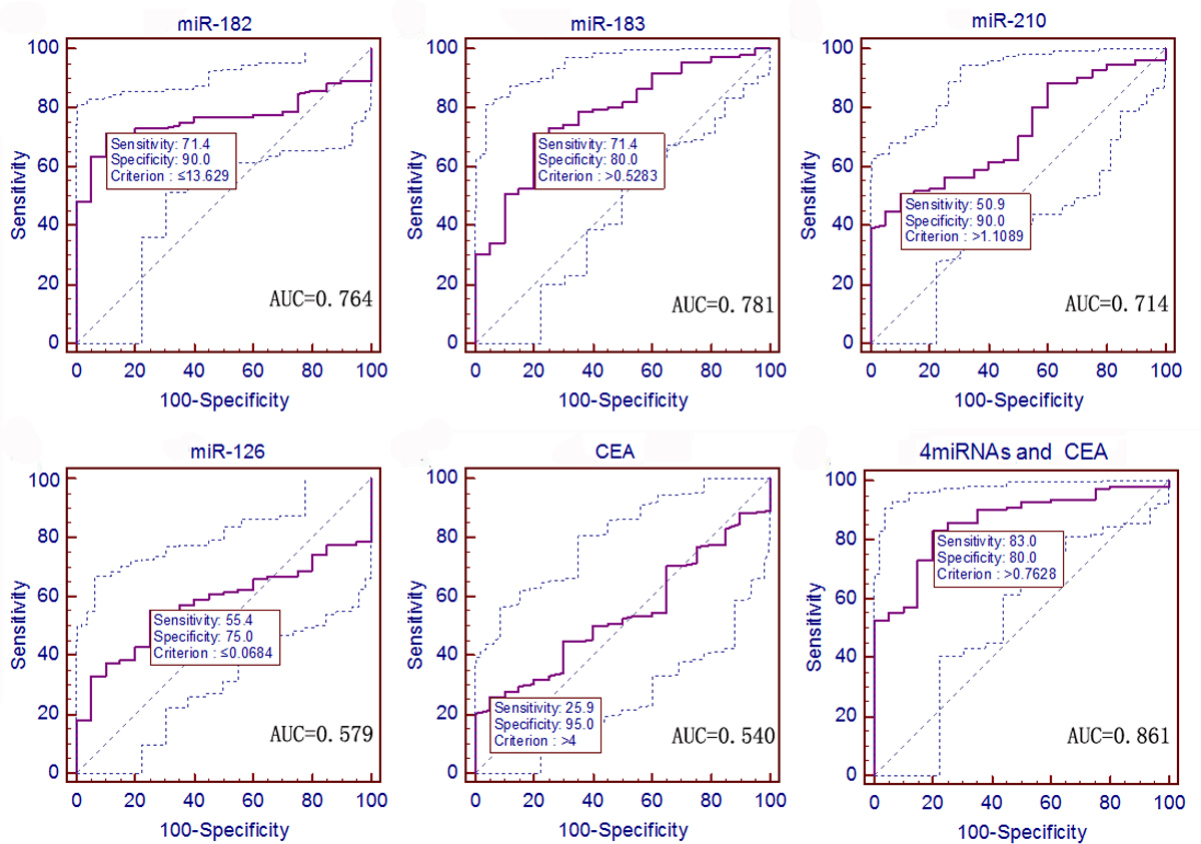
ROC curves to assess the value of serum miRNA and CEA levels in 112 NSCLC patients compared to 20 smokers. The *P* values of serum miR-182, miR-183, miR-210, miR-126, and CEA as well as the predictive value of logistic regression were *<* 0.0001, < 0.0001, 0.0001, 0.1322, 0.5053, and < 0.0001, respectively.

The ROC curves were constructed to evaluate the diagnostic value of the relative expression (2^-ΔΔCt^) of these four miRNAs plus CEA in the diagnosis of NSCLC. The data showed that the levels of miR-182, miR-183, and miR-210 were significantly distinct between NSCLC patients and tobacco smokers, with AUCs of 0.764, 0.781, and 0.714; sensitivities of 71.4%, 71.4%, and 50.9%; and specificities of 90.0%, 80.0%, and 90.0%, respectively (*P <* 0.0001, *<* 0.0001, and 0.0001; 95% CI: 0.683–0.834, 0.701–0.848, and 0.629–0.789; Fig 5), whereas the levels of miR126 and CEA had no obvious difference in AUCs to distinguish NSCLC and tobacco smokers (*P* = 0.1322, 0.5053; Fig 5). Moreover, the combination of these four miRNAs and CEA had an AUC of 0.861, with a sensitivity of 83.0% and a specificity of 80.0% (*P <* 0.0001, 95% CI: 0.790–0.915; Fig 5 and S2 Table).

In early-stage NSCLC, the data showed that the levels of miR-182, miR-183, and miR-210 were significantly distinct between NSCLC patients and tobacco smokers, with AUCs of 0.728, 0.787, and 0.748; sensitivities of 67.8%, 74.7%, and 55.2%; and specificities of 90.0%, 80.0%, and 90.0%, respectively (*P <* 0.0001, *<* 0.0001, and *<* 0.0001; 95% CI: 0.634–0.810, 0.698–0.861, and 0.655–0.827; S1 Fig), whereas the levels of miR-126 and CEA had no obvious difference in AUCs to distinguish NSCLC and tobacco smokers (*P* = 0.1205, 0.4240; S1 Fig). Moreover, the combination of these four miRNAs plus CEA had an AUC of 0.842, with a sensitivity of 86.2% and a specificity of 70.0% (*P <* 0.0001, 95% CI: 0.759–0.905; S1 Fig and S2 Table).

**Fig 6:**
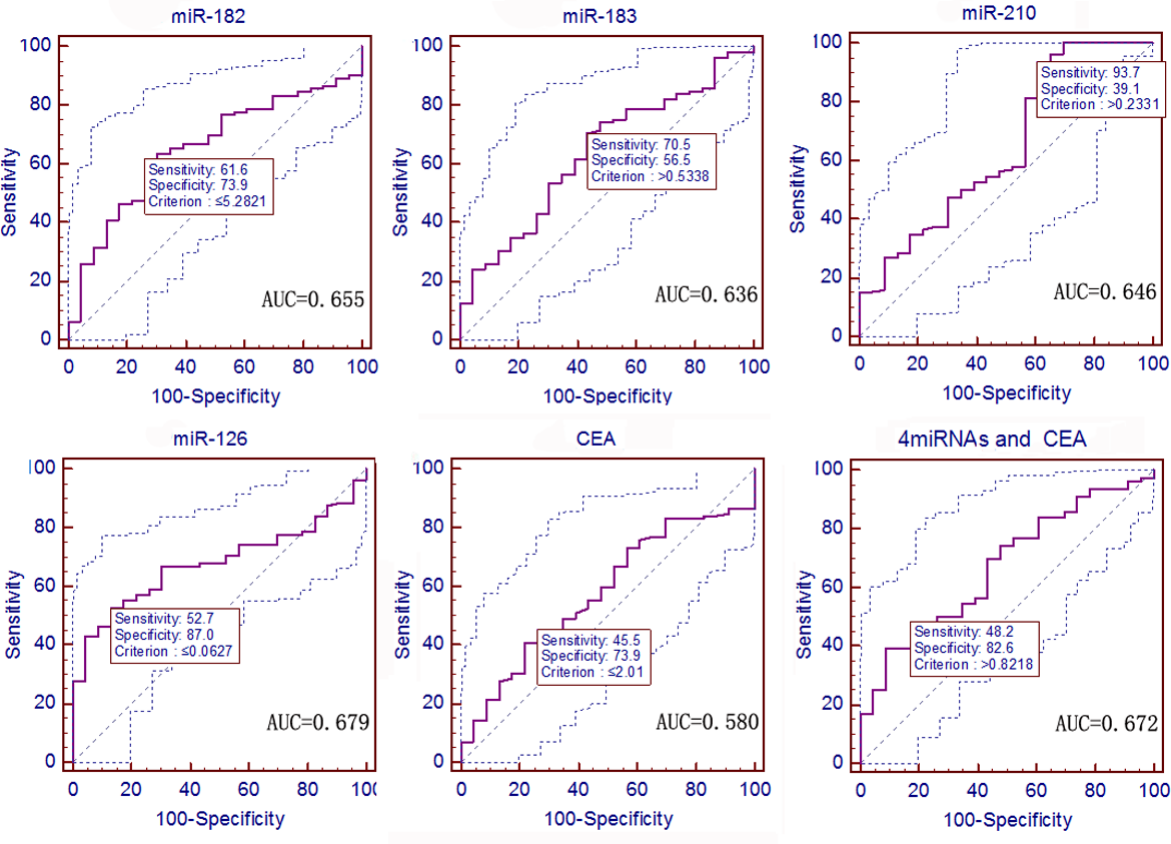
ROC curves to assess the value of serum miRNA and CEA levels in 112 NSCLC patients compared to 23 pneumonia patients. The *P* values of serum miR-182, miR-183, miR-210, miR-126, and CEA as well as the predictive value of logistic regression were 0.0061, 0.0259, 0.0350, 0.0002, 0.1914, and 0.0031, respectively.

Furthermore, the ROC curves revealed that the levels of serum miR-182, miR-183, miR-210, and miR-126 were able to distinguish NSCLC from pneumonia, with AUCs of 0.655, 0.636, 0.646, and 0.679; sensitivities of 61.6%, 70.5%, 93.8%, and 52.7%; and specificities of 73.9%, 56.5%, 39.1%, and 87.0%, respectively (*P* = 0.0061, 0.0259, 0.0350, 0.0002; 95% CI: 0.568–0.735, 0.549–0.717, 0.559–0.726, 0.593–0.756; Fig 6). However, the CEA level had no obvious difference in AUCs to distinguish NSCLC and pneumonia (*P* = 0.1914; Fig 6). Moreover, the diagnostic value of the combination of the four miRNAs plus CEA had an AUC of 0.861, with a sensitivity of 48.2% and a specificity of 82.6%, to distinguish NSCLC from pneumonia (*P* = 0.0031, 95% CI: 0.586–0.750; Fig 6 and S3 Table).

In early-stage NSCLC, the data showed that the levels of miR-182, miR-183, miR-210, miR126a, and CEA produced AUCs of 0.618, 0.648, 0.667, 0.672, and 0.676, with sensitivities of 56.3%, 78.2%, 94.3%, 56.3%, and 56.3% and specificities of 73.9%, 52.2%, 39.1%, 87.0%, and 73.9%, respectively, to distinguish NSCLC from pneumonia (*P* = 0.0487, 0.0182, 0.0145, 0.0006, and 0.0059; 95% CI: 0.521–0.709, 0.552–0.737, 0.571–0.754, 0.576–0.759, and 0.580–0.762; S2 Fig). The combination of these four miRNAs plus CEA produced an AUC of 0.731, with a sensitivity of 62.1% and a specificity of 73.9% (*P <* 0.0001, 95% CI: 0.638–0.811; S2 Fig and S3 Table).

**Fig 7:**
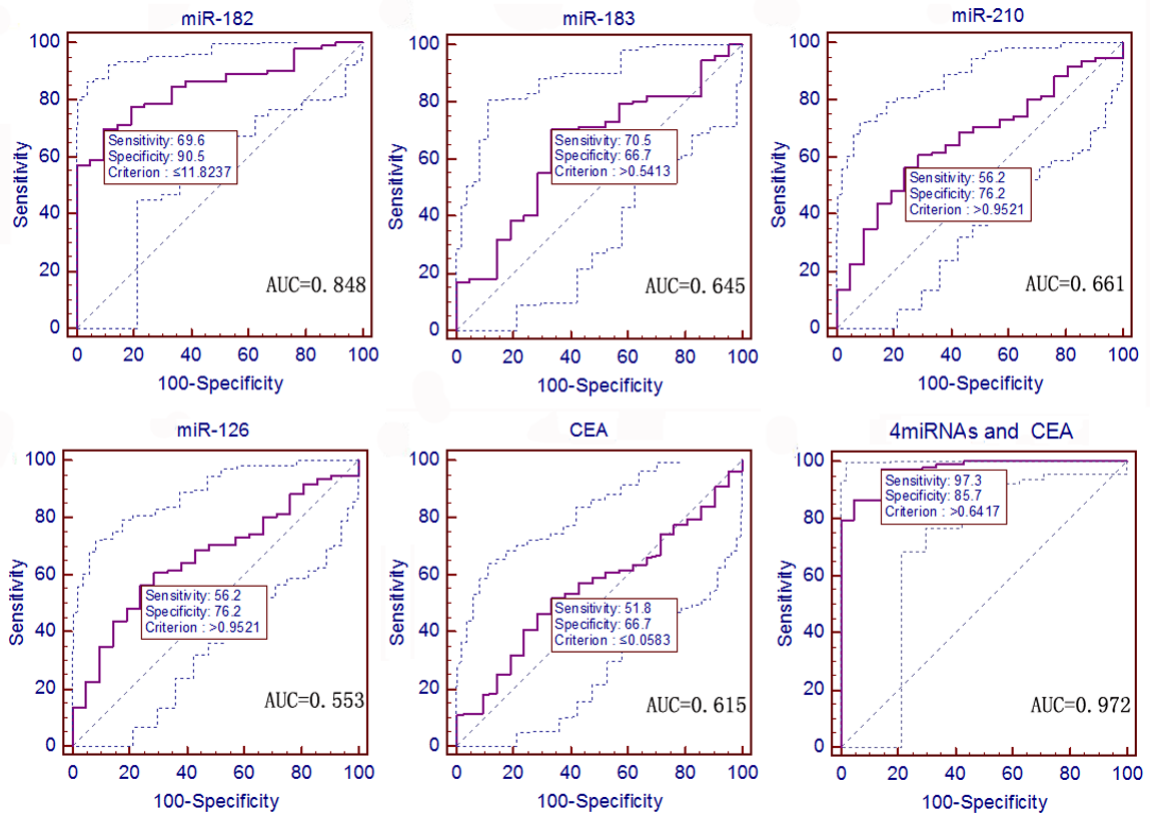
ROC curves to assess the value of serum miRNA and CEA levels in 112 NSCLC patients compared to 21 gastric cancer patients. The *P* values of serum miR-182, miR-183, miR-210, miR-126, and CEA as well as the predictive value of logistic regression were < 0.0001, 0.0241, 0.0063, 0.3961, 0.0744, and < 0.0001, respectively.

We also analyzed the AUCs between NSCLC and gastric cancer, i.e., miR-182, miR-183, and miR-210 showed AUCs of 0.848, 0.645, and 0.661, with sensitivities of 69.6%, 70.5%, and 56.3% and specificities of 90.5%, 66.7%, and 76.2%, respectively, to distinguish NSCLC from gastric cancer (*P <* 0.0001, 0.0241, and 0.0063; 95% CI: 0.775–0.904, 0.558–0.726, and 0.574–0.741, respectively; Fig 7). The combination of these four miRNAs plus CEA showed an AUC of 0.972, a sensitivity of 97.3%, and a specificity of 85.7% to distinguish NSCLC from gastric cancer (*P <* 0.0001; 95% CI: 0.928–0.993; Fig 7 and S4 Table).

In early-stage NSCLC, the ROC curves showed miR-182, miR-183, miR-210, and CEA with AUCs of 0.826, 0.660, 0.699, and 0.706; sensitivities of 66.7%, 73.6%, 66.7%, and 83.9%; and specificities of 90.5%, 66.7%, 71.4%, and 57.1%, respectively, to distinguish NSCLC from gastric cancer (*P <* 0.0001, = 0.0150, 0.0009, and 0.0016; 95% CI: 0.741–0.892, 0.562–0.748, 0.603–0.783, and 0.611–0.790, respectively; S3 Fig). The combination of these four miRNAs and CEA showed an AUC of 0.976, a sensitivity of 94.3%, and a specificity of 90.5% to distinguish NSCLC from gastric cancer (*P <* 0.0001; 95% CI: 0.927–0.996; S3 Fig and S4 Table).

### Serum miRNAs and NSCLC Clinical Pathology

In terms of the clinical pathology of NSCLC, we found no significant association between the serum levels of the four miRNAs and tobacco smoking status, lymph node metastasis, histological subtype of the tumor, tumor size, or pathological stage (*P >* 0.05; data not shown).

## Discussion

Serum miRNAs are useful as novel tumor biomarkers for various types of human cancer, but a significant variation exists in the data among different studies of the same disease [23]. The variation is largely attributed to different study populations, lack of standard endogenous controls for the miRNAs studied [24], age, limited sample sizes [25], and functional diversity of miRNAs [26]. Therefore, validation of serum miRNA levels is essential for future application in disease diagnosis. Furthermore, previous studies have focused on the diagnostic value of circulating miRNAs between NSCLC patients and healthy controls [14, 19, 25]. However, few studies have reported on the diagnostic value of circulating miRNAs between NSCLC patients and smokers, pneumonia patients, or other those with other malignancies. Thus, in the current study, we detected the serum levels of miR-182, miR-183, miR-210, and miR-126 in NSCLC patients in light of our previous studies and published literature [9, 13, 14, 22]. Our results demonstrated that these four miRNAs are potential tumor biomarkers for the diagnosis of NSCLC. Specifically, we found that the levels of miR-182, miR-183, and miR-210 were significantly increased in the sera from NSCLC patients, whereas the miR-126 levels were significantly decreased in the sera from NSCLC patients, compared to the healthy controls. The ROC curve analyses revealed that miR-182, miR-183, miR-210, or miR-126 alone was useful as a tumor biomarker for the early detection of NSCLC, with a high sensitivity and specificity. The combination of these four miRNAs with CEA further increased the diagnostic value, with an AUC of 0.965 (sensitivity of 81.3%, specificity of 100.0%, and accuracy of 90.8%). Furthermore, the data also showed that the relative expression levels of serum miR-182, miR-183, miR-210, and miR-126 enabled differentiation of NSCLC or early-stage NSCLC patients from current smokers as well as from patients with pneumonia or gastric cancer, with a high sensitivity and specificity. In conclusion, our current data indicate that the four serum miRNAs could serve as tumor biomarkers for the early detection of NSCLC.

To the best of our knowledge, our study is the first to report the use of combined serum miR-182, miR-183, miR-210, and miR-126 levels to differentiate early-stage NSCLC from healthy controls, current smokers, and pneumonia or gastric cancer patients. Indeed, individually, a previous study has shown that the serum levels of miR-182 and miR-183 were elevated in patients with NSCLC, compared to healthy controls [13]. Both miR-182 and miR-183 belong to the miR-183 family and act as onco-miRNAs in human tumorigenesis via the promotion of tumor cell growth and migration by targeting the transcription factor early growth response protein 1 [27, 28]. A previous meta-analysis has revealed that the expression of miR-183 family members is upregulated in many types of human cancer, in which NSCLC is ranked in third place [29]. Our results are consistent with previous studies demonstrating that the expression of miR-182 and miR-183 is increased not only in lung tumor tissues but also in the sera of NSCLC patients [13]. Zheng *et al*. [30] have demonstrated that plasma miR-182 is significantly increased in NSCLC patients, but it failed to distinguish stage I NSCLC from cancer-free controls. In addition, Abd-El-Fattah *et al*. [31] have shown that miR-182 is elevated in patients with NSCLC or pneumonia. However, in contrast to our results indicating that the serum miR-126 and miR-183 levels were altered between stage 0 and I NSCLC, Lin *et al*. [32] have shown significant differences in serum miR-126 and miR-183 levels between stage IV NSCLC patients and controls; whereas in stage I/II NSCLC patients, the miR-126 and miR-183 levels were stable and consistent. The reason for this discrepancy is unknown, but it may be related to the limited sample sizes and study populations. Furthermore, the level of miR-126 was downregulated in NSCLC compared to noncancerous lung tissues due to the fact that miR-126 participates in tumor angiogenesis by targeting the expression of vascular endothelial growth factor-A [33, 34]. Upregulated miR-126 also inhibits tumor cell adhesion, migration, and invasion, which may partially mediate Crk expression [35]. In our study, we showed that miR-126 was downregulated in sera from NSCLC patients, consistent with a previous study [15]. In addition, miR-210 has been called “the micromanager of the hypoxia pathway” and is involved in tumor growth, apoptosis, and angiogenesis [36]. Moreover, miR-210 has been reported to be increased in lung tumor tissues and sputum from NSCLC patients [37, 38]. In the current study, we demonstrated upregulation of serum miR-210 in NSCLC patients, which may act as an onco-miRNA in NSCLC development.

Although analysis of these four miRNAs has been reported previously, their diagnostic value to distinguish NSCLC from other diseases or conditions, such as current tobacco smoking, pneumonia, or other types of cancer, has not been evaluated to date. Our current study revealed that the levels of miR-182, miR-183, and miR-210 in NSCLC or early-stage NSCLC were significantly different from those of tobacco smokers as well as patients with pneumonia or gastric cancer, and they showed a high sensitivity and specificity. The serum level of miR-126 was able to distinguish NSCLC from pneumonia, whereas serum CEA levels had no such ability to distinguish NSCLC from other diseases. Thus, these four miRNAs might have the potential to be further evaluated as biomarkers for the early diagnosis of NSCLC. Moreover, the combination of these four miRNAs with CEA markedly increased the AUC value, with a high sensitivity and specificity, suggesting their potential role in the early diagnosis of NSCLC. Furthermore, previous studies have shown that these four miRNAs are differentially expressed in the sera of other diseases individually, but there has been no single study that reports the use of their combination as a biomarker for a disease. A few examples of these miRNAs in the literature are as follows: Li *et al*. [39] have shown that the serum levels of miR-182, miR-183, and miR-210 were higher in pregnant women with preeclampsia in their third trimester of pregnancy, compared to controls. The serum level of miR-182 was elevated in patients with hepatocellular carcinoma [40], breast cancer [41], or pulmonary tuberculosis, but not in those with pneumonia [31]. The circulating mirR-183 level was higher in patients with hepatocellular carcinoma [42] or tuberculosis [43]. In addition, the serum miR-210 level was also higher in various cancer patients, including those with acute myeloid leukemia [44], renal cell carcinoma [45], or bladder cancer [46]. Moreover, the serum level of miR-126 was downregulated in patients with malignant mesothelioma [47] or intracerebral hemorrhage [15]. Thus, after further confirmation, these four miRNAs could be useful for the early diagnosis of NSCLC. Additionally, the combination of these four miRNAs with the CEA level could reach a higher sensitivity and specificity for NSCLC diagnosis.

Our current data showed the diagnostic value of these four miRNAs to identify NSCLC, but previous studies reported by Markou *et al*. and Chen *et al*. have demonstrated otherwise [9, 14]. Specifically, Markou *et al*. have shown that miR-21 is significantly upregulated and that miR-126*, miR-30d, miR-30e-5p, and miR-451 are downregulated in NSCLC tissues; whereas there were differential levels of miR-21, miR-10a, and miR-30e-5p in the plasma, compared to those from healthy donors [9]. In addition, Chen *et al*. have identified 10 differentially expressed miRNAs in the sera from NSCLC patients, which was different from the miRNA profile of our current data. In our current study, we selected the four miRNAs according to previous studies from others and our own [9, 13, 15, 22]. Moreover, our current study had a few limitations related to small sample sizes as well as a lack of comparative analysis with malignancies other than gastric cancer. Thus, future studies with a larger sample size will be needed to confirm the benefit of combining the four miRNAs analyzed in this study for the early detection of NSCLC subtypes.

In conclusion, our study highlights the potential role of serum-circulating miR-182, miR-183, miR-210, and miR-126 levels for the early diagnosis of human primary NSCLC. The combination of these miRNAs with or without CEA enables clinicians to distinguish early-stage NSCLC patients from healthy controls, current smokers, as well as patients with pneumonia or gastric cancer.

## Supporting Information

S1 Fig ROC curves to assess the value of serum miRNA and CEA levels in 87 stage 0 and I NSCLC patients compared to 20 smokers. The *P* values of the serum level of miR-182, miR-183, miR-210, miR-126, and CEA as well as the predictive value of logistic regression were < 0.0001, < 0.0001, < 0.0001, 0.1205, 0.4240, and < 0.0001, respectively.
(TIF)

S2 Fig ROC curves to assess the value of serum miRNA and CEA levels in 87 stage 0 and I NSCLC patients compared to 23 pneumonia patients. The *P* values of the serum level of miR-182, miR-183, miR-210, miR-126, and CEA as well as the predictive value of logistic regression were 0.0487, 0.0182, 0.0145, 0.0006, 0.0059, and < 0.0001, respectively.
(TIF)

S3 Fig ROC curves to assess the value of serum miRNA and CEA levels in 87 stage 0 and I NSCLC patients compared to 21 gastric cancer patients. The *P* values of the serum level of miR-182, miR-183, miR-210, miR-126, and CEA as well as the predictive value of logistic regression were < 0.0001, 0.0150, 0.0009, 0.3302, 0.0016, and < 0.0001, respectively.
(TIF)

S1 Table Logistic regression analysis of the four miRNAs and CEA for the diagnosis of NSCLC or early-stage NSCLC. (DOCX)

S2 Table Sensitivity, specificity, and AUC of the four miRNAs and CEA in patients with NSCLC or early-stage NSCLC vs. smokers (2^-ΔΔCt^). (DOCX)

S3 Table Sensitivity, specificity, and AUC of the four miRNAs and CEA in patients with NSCLC or early-stage NSCLC vs. pneumonia (2^-ΔΔCt^). (DOCX)

S4 Table Sensitivity, specificity, and AUC of the four miRNAs and CEA in patients with NSCLC or early-stage NSCLC vs. gastric cancer (2^-ΔΔCt^). (DOCX)
