# Supplementary figures and images for: Diagnostic Value of Serum miR-182, miR-183, miR-210, and miR-126 Levels in Patients with Early-Stage Non-Small Cell Lung Cancer
Source: PLoS One. 2016 Apr 19;11(4):e0153046. doi: 10.1371/journal.pone.0153046 (PMC4836744; doi:10.1371/journal.pone.0153046)

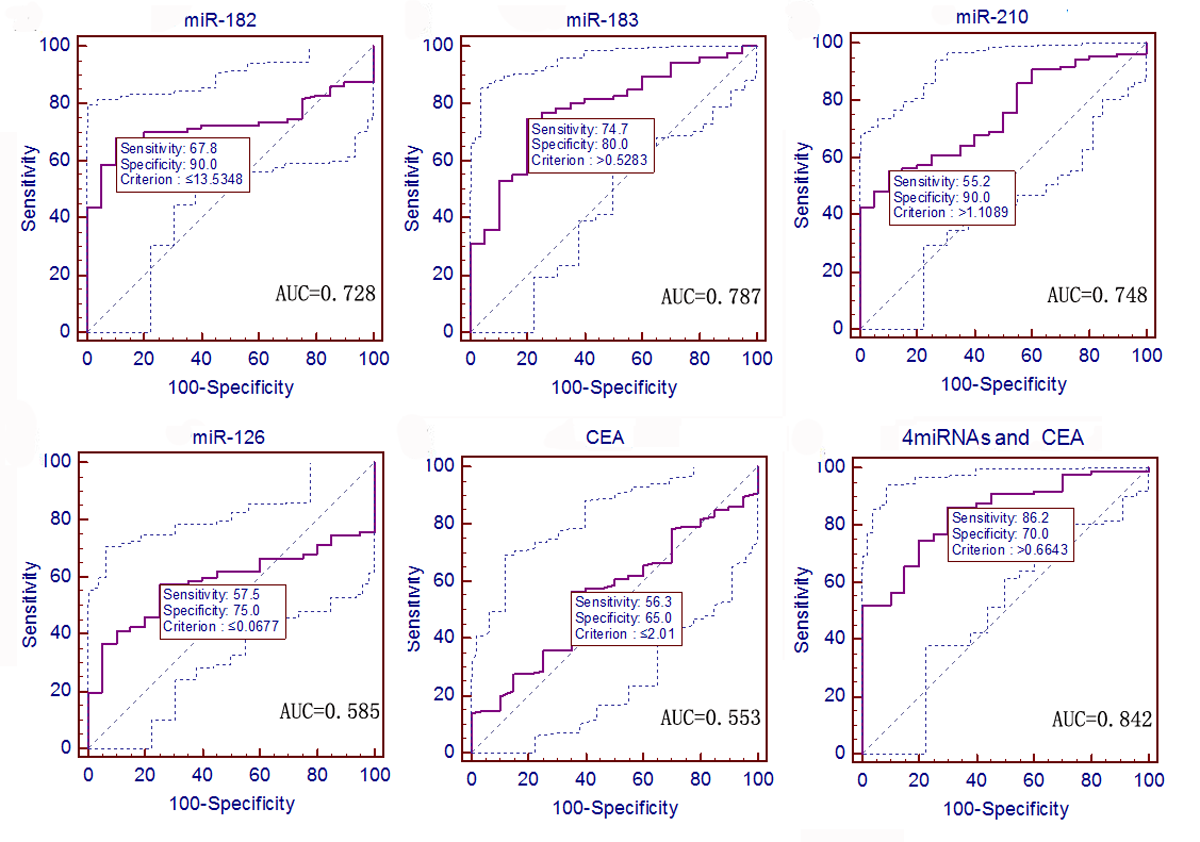

Supplement: S1 Fig — The P values of the serum level of miR-182, miR-183, miR-210, miR-126, and CEA as well as the predictive value of logistic regression were < 0.0001, < 0.0001, < 0.0001, 0.1205, 0.4240, and < 0.0001, respectively. (TIF) [file pone.0153046.s001.tif]

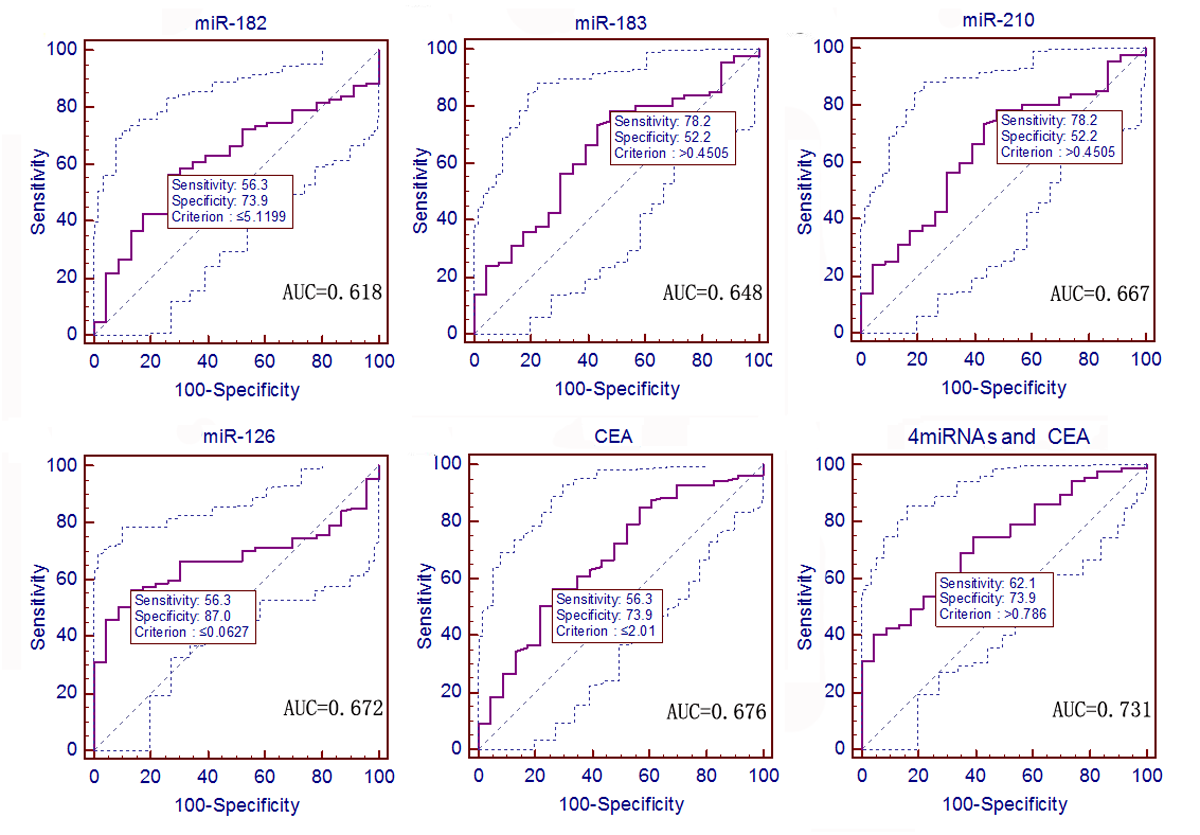

Supplement: S2 Fig — The P values of the serum level of miR-182, miR-183, miR-210, miR-126, and CEA as well as the predictive value of logistic regression were 0.0487, 0.0182, 0.0145, 0.0006, 0.0059, and < 0.0001, respectively. (TIF) [file pone.0153046.s002.tif]

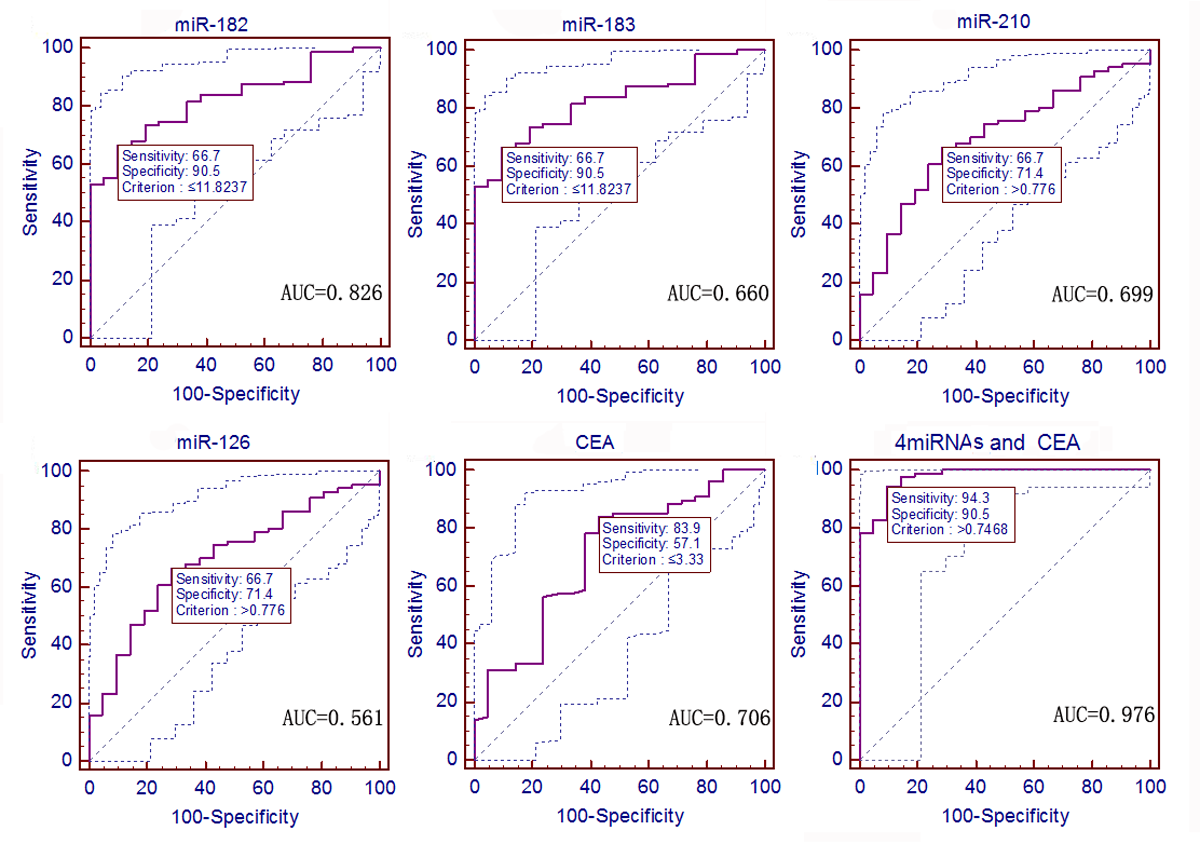

Supplement: S3 Fig — The P values of the serum level of miR-182, miR-183, miR-210, miR-126, and CEA as well as the predictive value of logistic regression were < 0.0001, 0.0150, 0.0009, 0.3302, 0.0016, and < 0.0001, respectively. (TIF) [file pone.0153046.s003.tif]
