# Supplementary material for: Diagnostic Value of Serum miR-182, miR-183, miR-210, and miR-126 Levels in Patients with Early-Stage Non-Small Cell Lung Cancer
Source: PLoS One. 2016 Apr 19;11(4):e0153046. doi: 10.1371/journal.pone.0153046 (PMC4836744; doi:10.1371/journal.pone.0153046)
Supplement: S1 Table — (DOCX) [file pone.0153046.s004.docx]

**S1 Table. Logistic regression analysis of four miRNAs and CEA in the diagnosis of NSCLC or early-stage NSCLC**

| Variable | Coefficient | *P* value | Odds ratio | 95% CI |
| --- | --- | --- | --- | --- |
| NSCLC |  |  |  |  |
| miR-182 | 145.08696 | 0.0006 | 0.4013 | 0.2661–0.6050 |
| miR-183 | 107.31153 | 0.2953 | 1.02E+63 | 1.59368E+27–658.49883E+96 |
| miR-210 | 13.17854 | 0.2449 | 40.3E+45 | 2.08706E-41–77.6412E+132 |
| miR-126 | -0.91317 | < 0.0001 | 529E+3 | 0.0001–2.34E+15 |
| CEA | 1.35630 | 0.0007 | 3.8818 | 1.7689–8.5185 |
| Constant | -2.8395 |  |  |  |
| Early-stage NSCLC |  |  |  |  |
| miR-182 | 162.22875 | 0.0031 | 28.5E+69 | 519.36354E+21–1.5654E+117 |
| miR-183 | 115.27682 | 0.3934 | 116E+48 | 1.24101E-65–1.08254E+165 |
| miR-210 | 5.47361 | 0.6758 | 238.3182 | 1.72845E-9–32.9E+12 |
| miR-126 | -0.87309 | 0.0002 | 0.4177 | 0.2625–0.6646 |
| CEA | 1.50240 | 0.0027 | 4.4925 | 1.6807–12.0084 |
| Constant | -2.6067 |  |  |  |
