# Supplementary material for: Diagnostic Value of Serum miR-182, miR-183, miR-210, and miR-126 Levels in Patients with Early-Stage Non-Small Cell Lung Cancer
Source: PLoS One. 2016 Apr 19;11(4):e0153046. doi: 10.1371/journal.pone.0153046 (PMC4836744; doi:10.1371/journal.pone.0153046)
Supplement: S2 Table — (DOCX) [file pone.0153046.s005.docx]

**S2 Table. Sensitivity, specificity, and AUC of four miRNAs and CEA in the diagnosis of NSCLC or early-stage NSCLC, compared to tobacco smokers (2^-ΔΔCt^)**

| Potential tumor marker | Cut-off value | Sensitivity (%) | Specificity (%) | AUC (95% CI) | *P* |
| --- | --- | --- | --- | --- | --- |
| NSCLC |  |  |  |  |  |
| miR-182 | 13.629 | 71.4 | 90.0 | 0.764 (0.683–0.834) | < 0.0001 |
| miR-183 | 0.5283 | 71.4 | 80.0 | 0.781 (0.701–0.848) | < 0.0001 |
| miR-210 | 1.1089 | 50.9 | 90.0 | 0.714 (0.629–0.789) | 0.0001 |
| miR-126 | 0.0684 | 55.4 | 75.0 | 0.579 (0.490–0.664) | 0.1322 |
| CEA | 4.0 | 25.9 | 95.0 | 0.540 (0.451–0.627) | 0.5053 |
| The four miRNAs + CEA | 0.7628 | 83.0 | 80.0 | 0.861 (0.790–0.915) | < 0.0001 |
| Early-stage NSCLC |  |  |  |  |  |
| miR-182 | 13.5348 | 67.8 | 90.0 | 0.728 (0.634–0.810) | < 0.0001 |
| miR-183 | 0.5283 | 74.7 | 80.0 | 0.787 (0.698–0.861) | < 0.0001 |
| miR-210 | 1.1089 | 55.2 | 90.0 | 0.748 (0.655–0.827) | < 0.0001 |
| miR-126 | 0.0677 | 57.5 | 75.0 | 0.585 (0.486–0.680) | 0.1205 |
| CEA | 2.01 | 56.3 | 65.0 | 0.553 (0.454–0.649) | 0.4240 |
| The four miRNAs + CEA | 0.6643 | 86.2 | 70.0 | 0.842 (0.759–0.905) | < 0.0001 |
