# Supplementary material for: Diagnostic Value of Serum miR-182, miR-183, miR-210, and miR-126 Levels in Patients with Early-Stage Non-Small Cell Lung Cancer
Source: PLoS One. 2016 Apr 19;11(4):e0153046. doi: 10.1371/journal.pone.0153046 (PMC4836744; doi:10.1371/journal.pone.0153046)
Supplement: S3 Table — (DOCX) [file pone.0153046.s006.docx]

**S3 Table. Sensitivity, specificity, and AUC of four miRNAs and CEA in the diagnosis of NSCLC or early-stage NSCLC, compared to pneumonia (2^-ΔΔCt^)**

| Potential tumor marker | Cut-off value | Sensitivity (%) | Specificity (%) | AUC (95% CI) | *P* |
| --- | --- | --- | --- | --- | --- |
| NSCLC |  |  |  |  |  |
| miR-182 | 5.2821 | 61.6 | 73.9 | 0.655 (0.568–0.735) | 0.0061 |
| miR-183 | 0.5338 | 70.5 | 56.5 | 0.636 (0.549–0.717) | 0.0259 |
| miR-210 | 0.2331 | 93.8 | 39.1 | 0.646 (0.559–0.726) | 0.0350 |
| miR-126 | 0.0627 | 52.7 | 87.0 | 0.679 (0.593–0.756) | 0.0002 |
| CEA | 2.01 | 45.5 | 73.9 | 0.580 (0.580–0.664) | 0.1914 |
| The four miRNAs + CEA | 0.8218 | 48.2 | 82.6 | 0.672 (0.586–0.750) | 0.0031 |
| Early-stage NSCLC |  |  |  |  |  |
| miR-182 | 5.1199 | 56.3 | 73.9 | 0.618 (0.521–0.709) | 0.0487 |
| miR-183 | 0.4505 | 78.2 | 52.2 | 0.648 (0.552–0.737) | 0.0182 |
| miR-210 | 0.2331 | 94.3 | 39.1 | 0.667 (0.571–0.754) | 0.0145 |
| miR-126 | 0.0627 | 56.3 | 87.0 | 0.672 (0.576–0.759) | 0.0006 |
| CEA | 2.01 | 56.3 | 73.9 | 0.676 (0.580–0.762) | 0.0059 |
| The four miRNAs + CEA | 0.786 | 62.1 | 73.9 | 0.731 (0.638–0.811) | < 0.0001 |
