# Supplementary material for: Diagnostic Value of Serum miR-182, miR-183, miR-210, and miR-126 Levels in Patients with Early-Stage Non-Small Cell Lung Cancer
Source: PLoS One. 2016 Apr 19;11(4):e0153046. doi: 10.1371/journal.pone.0153046 (PMC4836744; doi:10.1371/journal.pone.0153046)
Supplement: S4 Table — (DOCX) [file pone.0153046.s007.docx]

**S4 Table. Sensitivity, specificity, and AUC of four miRNAs and CEA in the diagnosis of NSCLC or early-stage NSCLC, compared to gastric cancer (**2^-ΔΔCt^**)**

| Potential tumor marker | Cut-off value | Sensitivity (%) | Specificity (%) | AUC (95% CI) | *P* |
| --- | --- | --- | --- | --- | --- |
| NSCLC |  |  |  |  |  |
| miR-182 | 11.8237 | 69.6 | 90.5 | 0.848 (0.775–0.904) | <0.0001 |
| miR-183 | 0.5413 | 70.5 | 66.7 | 0.645 (0.558–0.726) | 0.0241 |
| miR-210 | 0.9521 | 56.3 | 76.2 | 0.661 (0.574–0.741) | 0.0063 |
| miR-126 | 0.0583 | 51.8 | 66.7 | 0.553 (0.465–0.640) | 0.3961 |
| CEA | 3.33 | 71.4 | 57.1 | 0.615 (0.527–0.698) | 0.0744 |
| The four miRNAs + CEA | 0.8304 | 97.3 | 85.7 | 0.972 (0.928–0.993) | <0.0001 |
| Early-stage NSCLC |  |  |  |  |  |
| miR-182 | 11.8237 | 66.7 | 90.5 | 0.826 (0.741–0.892) | <0.0001 |
| miR-183 | 0.5413 | 73.6 | 66.7 | 0.660 (0.562–0.748) | 0.0150 |
| miR-210 | 0.776 | 66.7 | 71.4 | 0.699 (0.603–0.783) | 0.0009 |
| miR-126 | 0.0494 | 50.6 | 71.4 | 0.561 (0.462–0.656) | 0.3302 |
| CEA | 3.33 | 83.9 | 57.1 | 0.706 (0.611–0.790) | 0.0016 |
| The four miRNAs + CEA | 0.7468 | 94.3 | 90.5 | 0.976 (0.927–0.996) | < 0.0001 |
